# The Zagreb Collection of human brains: entering the virtual world

**DOI:** 10.3325/10.3325/cmj.2018.59.283

**Published:** 2018-12

**Authors:** Pero Hrabač, Ana Bosak, Mario Vukšić, Miloš Judaš, Ivica Kostović, Željka Krsnik

**Affiliations:** 1 Department of Medical Statistics, Epidemiology, and Medical Informatics, “Andrija Štampar” School of Public Health, University of Zagreb School of Medicine, Zagreb, Croatia *phrabac@hiim.hr*; 2 Croatian Institute for Brain Research, Center of Research Excellence for Basic, Clinical and Translational Neuroscience, University of Zagreb School of Medicine, Zagreb, Croatia

The Zagreb Collection of human brains was founded at the University of Zagreb School of Medicine in the early 1970s by Professor Ivica Kostović. Over the last 40 years, the Collection has been augmented with over 100 000 histological slides of the brain tissue (1,2). The tissue samples were obtained from more than 1300 human brains in all stages of development, including fetal and early postnatal period, both from healthy individuals and individuals with developmental, psychiatric, and neurological conditions (3-11).

In the early days, while the Collection consisted of a few dozen brains, with blocks and histologic specimens obtained (cut) from these brains, classification of its contents was easily solvable by a simple card catalog. With the collection content growing, paper-based solution has become obsolete. The idea about computer-based catalog with real-time access to specimen images digitized in high resolution appeared in the early 1990s. However, more than 20 years had to pass before the first catalog became functional. In this article, we describe the hardware, software, and logistic problems we have encountered along the way, as well as the final online software solution to this problem.

## History

Even though the Collection beginnings were humble, from 1974 until today there has been a constant inflow of source material and histological slides. In this context, the first problem we had to solve was the physical space necessary to store this type of collection. This was finally solved in 1998 by moving the Collection from the old Department of Anatomy building into the newly founded Croatian Institute for Brain Research.

On the purely theoretical level, the task of making a computer-based collection catalog seemed relatively simple. All we needed to do was to create a relational computer database in which the “parental” element is a macroslide, ie, the brain. From each brain, it is then possible to cut an arbitrary number of blocks, and from each block it is possible to obtain an arbitrary number of microscopic slides. In this relational model, each child object (ie, block) inherits parental characteristics (from the brain of its origin), such as age, sex, post-mortem time, possible pathology, and other attributes. Histological slide, by belonging to a certain block, inherits the number, location, date of processing, type of fixation used, but also all the attributes of the brain from which the block originated. This type of hierarchical data organization simplifies data search and ensures consistency of the information in the sense that every tissue sample must belong to a block and every block must belong to a brain.

The initial catalogization process was relatively straightforward, consisting basically of entering the data (brain and block characteristics) into an Excel table. However, digitizing the histological specimens was a few orders of magnitude more complicated. The first images of histological slides were digitized in 1994 by scanning on a flatbed scanner. The slides were first photographed using a camera with low-sensitivity, small grain 35mm professional Kodak film. Since the goal from the start has been to have a digital image of the entire specimen and not just the part of it that is normally visible through the microscope, specimens could not be filmed using the traditional method with the camera mounted on the microscope. To obtain the image of the whole specimen (ie, the whole glass with the histological material), the specimen was first mounted on a negatoscope. We used an Olympus 35mm camera with high-quality macro lenses to photograph the specimens. The camera was fixed at an appropriate distance from the specimen, so that the specimen took up the highest possible part of the viewing area, and consequently the largest part of the film. By experimenting with lenses (macro vs portrait vs wide angle lenses), types and sensitivity of the film (mostly 100 ASA or lower), and different camera setups, we obtained satisfying results. The film was developed to the A4 size (210 × 297 mm) on professional paper (Ilford, Knutsford, UK) and scanned at the highest resolution commercially available at the time, which was 300 dots per inch (dpi). Using a Microtek (Hsinchu, Taiwan) scanner, we obtained the first specimen images, with the size of approximately 3600 × 2400 pixels. This procedure, although devised more than 20 years ago and although based on a lengthy analogue-digital process, produced usable results even for today's standards. Moreover, while restoring 35mm film, we have recently found out that the maximum resolution of quality low-sensitive (50 ASA) film is about 6000 × 4000 pixels, which is in line with the declared granulation of these films of around 10 μm. So, by using the described process, we in fact came close to the physical limits of the film itself. However, this process clearly had many limitations. First and foremost, the lengthy process of film development did not allow us to get immediate insight into the material. Also, as with every system consisting of many elements, a certain quality loss was present in each step – lighting, lenses, camera quality, film grain, photographic paper, and the scanner used for digitizing the images. Although the process gave us usable results, soon we started to look for a new solution that would allow us to directly digitize histological specimens without the mediation of analog technology and the related loss of quality.

## Early approaches to digitalization

In our early digitizing attempts, we were mostly experimenting with a desktop (flatbed) scanners equipped with a transparency adapter. Such adapters were originally intended for scanning transparent materials (foils) for overhead projectors but were soon embraced by photographers for scanning strips or individual slides of 35mm negative or positive films. With some out-of-the-box thinking, we managed to scan the glass slides using the positive film settings of the scanner software. The main limiting factor at that time (mid- to late-1990s) was scanner resolution, which was seldom above 1200 dpi. This equals about 50 dots per millimeter, meaning that the smallest feature that can be observed on the specimens is around 1/50 mm, ie, 20 μm. Although this was significantly below our needs, the digitization speed and quality suggested that we were heading into the right direction. The time required for scanning one slide was reduced from day(s) to just a few minutes, with significantly higher quality of the final result. The only problem that needed to be overcome was scanner resolution.

The answer to our problems came from an unexpected direction. In 1997, the Nikon Company (Tokyo, Japan) produced its first series of small, cheap, and fast high-resolution (for that time) film-scanners. For a price of less than US $2000, we bought a Nikon Super CooLScan 1000 scanner, which could scan the entire 35mm slide just under 3 minutes at 2700 dpi resolution. Although this device was designed to scan 35mm film, by adjusting the plastic guides that held the film during the scanning (frankly, using anything from adhesive tape to wooden sticks) it was possible to directly scan the glass histological specimens. In subsequent years, with the increase in resolution to 4000, and later to 6400 dpi, the amount of detail in the final result has significantly increased, so that the specimens digitized in this way could be routinely used in our publications. Additionally, moving from 8 to 12, and later to 16-bit color depth, meaning that the number of colors recognized by the scanner grew from millions to billions, greatly improved color accuracy and delivered unprecedented digitization quality in a desktop format.

However, after the first few dozen slides were scanned in this way, it became clear that we were still faced with a number of problems. A specimen digitized using Nikon device at 2700 dpi resolution (amounting to approximately 3000 × 2500 pixels) took up about 25 Mb of disk space when stored in a lossless TIFF format. These files represented a serious challenge for both central processing unit and data storage. In other words, we managed to partly overcome the resolution problem but encountered the storage (disk) space problem. A possible solution was to group a number of disks in an array. Standard for redundant data storage, Redundant Arrays of Inexpensive Disks (RAID) technology, was at the time a relatively new and exotic solution based on rare and expensive RAID disk controllers. However, the mid 1990s saw the rise of data storage based on optical technology (compact discs, CDs), with arrival of the first recorders priced below US $1000. Although the CD technology was advertised as a solution for data storage for the next 100 years, already in the early 2000s many CD media were unreadable. Consequently, a large number of slides from this period was irretrievably lost due to problems with the early optical media.

Through this process, many lessons were learned and it became obvious that only feasible approach to digitizing the whole collection was a relational software solution, with separate database and image storage. Early solutions implemented in the DOS (Disk Operating System) environment on the Clipper platform (Nantucket, MA, USA) were sufficient for access to essential information about the specimens, but image storage and distribution presented too heavy load for the server and network infrastructure of the time. By switching to the Windows platform and hardware based on Intel Pentium III and early Pentium IV processors (Santa Clara, CA, USA), we implemented a number of solutions based on Delphi (Borland, Austin, TX, USA), Access (Microsoft, Redmond, WA, USA), and Filemaker (Apple, Cupertino, CA, USA) database software. Along with the increase in processing power and storage capacity, there also grew the average size of the scanned products. When a new generation of Nikon scanners with 4000 dpi resolution appeared, the size of an average specimen stored as TIFF image rose to 60-100 Mb. With the steady increase in scanner technology (primary in terms of resolution), we soon found ourselves in a vicious circle of technology progression, as we were scanning the same slides every 2 or 3 years on devices with ever-increasing resolution. While the quality of digitized samples increased, scanning time remained more or less the same, with 3-5 minutes needed to digitize each specimen. Taking into consideration the time needed to prepare (mount) the specimens for scanning and software post-processing, it was decided that the digitization project should be put aside until the technology allows us to significantly improve scanning resolution. We realized that the general approach with relational database model and separate storage solution was theoretically sound, but the technology needed to implement such a project was not sufficiently developed.

## Current solution

### Problem definition

The digitization project had to wait until a “balance” between quality of digitization devices, processor power, storage capacity, and bandwidth of computer networks was achieved. In addition, due to the budget constraints, all system elements had to be an in-house solution, which excluded customized software or hardware solutions.

Finally, around 2010, an answer to our needs began to emerge. On the hardware side, Intel Core i5 and Core i7 processors in combination with relatively cheap RAM have brought within our grasp multiprocessor systems with high clock speeds and 8 or 16 Gb RAM. Network bandwidth, with speeds of 1 Gbit or higher, enabled a remote data storage that can be simultaneously accessed from multiple remote systems. Devices for network-attached storage (NAS), especially ones from SOHO (small office/home office) segment such as Synology (Taipei, Taiwan), Western Digital (San Jose, CA, USA), and QNAP (New Taipei City, Taiwan) promised fast network access and redundancy in data storage, which was for the first time financially available to us. Finally, open-source software solutions based on the combination of Linux operating system, platforms for content creation, and database management have put all that power within our reach. Although only a few years ago our only hope was a custom-made software solution from third-party suppliers, we could now configure our own fast and highly customized solution for data storage and cataloguing.

### Implementation – a system for sample digitization

The cornerstone of the system was and remained a scanner. Native resolution of the first scanners we used to directly (without the film mediation) scan the slides was 600 dpi (dots or lines per inch), or about 236 dots (lines) per centimeter. Using a simple calculation one can obtain the theoretical maximum resolution of this digitization process: 10000 (micrometers in centimeter) / 236 (scanner resolution in lines per centimeter) = 42 μm. Due to primarily the quality of scanner optics, this maximum value was impossible to achieve in practice, but we could distinguish details from 50 to 60 μm. With further increase in optical resolution, it was possible to identify finer details – the size of approximately 15 μm (at 2700 dpi scanner resolution, circa 1997), 10 μm (at 4000 dpi, circa 2000), and 5 μm (6400 dpi, circa 2005). The latter values allowed us to recognize such elements as individual neurons and erythrocytes in the capillaries. Therefore, since the resolution was considered as the most important parameter for digitization of the Collection, we purchased a slide scanner Hamamatsu-Nanozoomer 2.0RS (Hamamatsu, Japan). Since the newer devices for digitization of microscopic samples do not work in exactly the same way as traditional scanners, resolution cannot be directly comparable. However, by combining the manufacturer's specifications with our practical experience, we found that the maximum resolution of this device is close to 40000 dpi at the highest magnification, allowing us to observe details smaller than 1 μm in diameter. Finally, a solution was available to digitize the specimens in resolution that, one could reasonably speculate, would eliminate future re-scanning of the same specimens using newer machines with even higher resolution.

### Implementation – hardware

We have based the hardware solution on HP 7200 Series desktop computer (HP, Palo Alto, CA, USA), with i7-2600 processor and nominal clock frequency of 3.4 GHz. The system was paired with 16 Gb of RAM and Samsung SSD 840 Pro Series hard drive (Samsung, Seoul, South Korea). This disk was used to install the operating system and other necessary software, while for the data (specimen) storage we used a NAS system (Synology, model DS1812+). To allow an additional level of data redundancy, NAS was configured in a RAID array type 6, with 8 Western Digital WD30EFRX RED hard drives for a total capacity of 18 Tb. RAID 6 configuration allows system to function normally after the failure of up to any two of the eight installed hard drives. Although RAID 6 configuration can cause a decline rate of speed transmission when writing (but not reading), this shortage has been accepted to achieve additional data security. Finally, the NAS device was connected to a local area network (speed of 1 Gbit) by a dedicated gigabit network switch (HP 1810-8 ProCurve).

This type of system allows considerable flexibility in the sense that all the elements can be expanded in accordance with the available funding. Also, data are protected from hardware failure by redundancy of RAID systems used for storage. The overall price of thus designed system was below €3000, making these and similar solutions accessible even in institutions with very limited budget. In addition, taking into account that this solution is based on open source software and does not require additional expenses for program support, we believe it is an interesting alternative to a system based on commercial software.

### Implementation – software

The hardware development over the past two decades has been a key factor in solving the problem of high-resolution digitization (scanning) and cataloguing of specimens in our Collection. Nevertheless, our ultimate goal was to create a software solution that will make full use of such development. Given the financial constraints, we decided to solve the problem by using in-house expertise and open source software. The specific solution is based on the Linux operating system, version Ubuntu 16.04 (Canonical Ltd, London, UK). Additionally installed on the operating system are Apache web server (Apache Software Foundation, Wakefield, MA, USA), a relational database management system MySql (Oracle Corporation, Redwood Shores, CA, USA), and a server-side scripting language PHP (Zend Technologies Ltd, Cupertino, CA, USA). These software technologies were merged by using Joomla content management system (Open Source Matters, Inc., New York, NY, USA) with a few additional plugins, most notably the Fabrik custom application building software (Media A-Team Inc., Houston, TX, USA).

Using the Joomla system, we created a website in order to implement a user-friendly interface enabling the access to the Collection contents from any computer worldwide. A relational database with the previously described hierarchical structure (brain-blocks-specimens) was created in MySql, while the Fabrik plugin for Joomla enabled the integration of this database into an easily manageable, web-accessible system. In this way, the users can access the database by using any commercially available web browser, ie, without the need to install any software on their PCs. An additional advantage is that the database can be accessed from any kind of PC, independently of its software platform (PC, Macintosh, or Linux) or even from Android- or iOS-based mobile phones.

The demo version of the database user interface can be accessed on the database website (*www.zagrebbraincollection.hr*), using login and password supplied on the page. The user can browse the database in a quick and secure manner by brain, block, or specimen, and using the attributes such as age, post-mortem time, sex, crown-rump length, diagnosis (for brain, inherited by blocks and specimens cut from that brain) or processing method, source brain, plane of section (for block, inherited by specimens cut from that block), or staining (for specimen). Besides information on brains and blocks, users can access scanned specimen images. The demo version includes only a small selection of low-resolution JPEG images, while the users registered with full privileges can access high-quality images (typically with >20 megapixel resolution) in a proprietary NDPI file format. This format offers some additional options, most notably files composed of multi-layered images, which allow the user to scroll up and down through subsequent tissue layers.

## Conclusion

As every major collection in natural sciences and elsewhere, the Zagreb Collection of Human Brains has met the problem of cataloguing its content. In the early days, the only possible solution to this problem was paper-based because the appropriate methods of digitizing data (primarily histological specimens) did not exist. Only after 2000, the rapid development of hardware and software platforms enabled the realization of the first functional solution that allows the end user to preview the Collection content and access the specimen images stored in digital format. Through the digitization project, we have made a significant step in facilitating access to the vast Collection content, but also devised an interesting system of digital cataloguing of a large number of high-resolution images. Such a system is potentially interesting in all areas of science where secure storage and rapid access to large data volume are needed, and the budget does not allow the use of third-party customized solutions.
